# Subtropical hibernation in juvenile tegu lizards (*Salvator merianae*): insights from intestine redox dynamics

**DOI:** 10.1038/s41598-018-27263-x

**Published:** 2018-06-19

**Authors:** Daniel C. Moreira, Alexis F. Welker, Élida G. Campos, Silvia Cristina R. de Souza, Marcelo Hermes-Lima

**Affiliations:** 1 Departamento de Biologia Celular, Universidade de Brasília, Brasília, 70910-900 Brazil; 2 Área de Morfologia, Faculdade de Medicina, Universidade de Brasília, Brasília, 70910-900 Brazil; 3 Faculdade da Ceilândia, Universidade de Brasília, Brasília, 72220-275 Brazil; 4 Departamento de Fisiologia, Instituto de Biociências, Universidade de São Paulo, São Paulo, 05508-090 Brazil

## Abstract

Juvenile tegu lizards (*Salvator merianae*) experience gradual and mild temperature changes from autumn to winter in their habitat. This tropical/subtropical reptile enter a state of dormancy, with an 80% reduction in metabolic rate, that remains almost constant during winter. The redox metabolism in non-mammalian vertebrates that hibernate under such distinguished conditions is poorly understood. We analyzed the redox metabolism in the intestine of juvenile tegus during different stages of their first annual cycle. The effect of food deprivation (in spring) was also studied to compare with fasting during hibernation. Both winter dormancy and food deprivation caused decreases in reduced glutathione levels and glutathione transferase activity. While glutathione peroxidase and glutathione transferase activities decreased during winter dormancy, as well as glutathione (GSH) levels, other antioxidant enzymes (catalase, superoxide dismutase and glutathione reductase) remained unchanged. Notably, levels of disulfide glutathione (GSSG) were 2.1-fold higher in late autumn, when animals were in the process of depressing metabolism towards hibernation. This increased “oxidative tonus” could be due to a disruption in NADPH-dependent antioxidant systems. In dormancy, GSSG and lipid hydroperoxides were diminished by 60–70%. The results suggest that the entrance into hibernation is the main challenge for the redox homeostasis in the intestine of juvenile tegus.

## Introduction

Large fluctuations in oxygen availability or consumption are associated with altered mitochondrial reactive oxygen species (ROS) production, which may cause oxidative stress, functional losses and death in animals^1,2^. In spite of that, many animals have the ability to seasonally enter into a state of metabolic depression and adapt to intense changes in oxygen availability or consumption. Despite the diversity of species that suppress their metabolism in response to harsh environments, most of them share some general physiological and molecular responses^3–5^. The modulation of endogenous antioxidants has been reported in animals that undergo freezing and hypoxic conditions, and during aestivation and hibernation, apparently being one of the common features of seasonal metabolic suppression phenomena^6–9^.

In such context, there is very little information regarding ROS management by endogenous antioxidants - i.e. the redox metabolism - during hibernation in non-mammalian vertebrates. Only a few investigations were done on the redox metabolism in hibernating amphibians and reptiles^10–18^. Moreover, for the best of our knowledge, there is no information on the redox metabolism during tropical hibernation. One organism that could fill this gap is the tegu lizard *Salvator merianae*, from South America, which has been well studied in terms of physiological and metabolic adjustments during tropical/subtropical hibernation^19–22^. Juvenile tegu lizards do not feed and become dormant almost continuously (although adults show some quick arousals^19,22^) for 4–5 months, during the dry winter season. De Souza and coworkers^20^ reported an 80% reduction in metabolic rate of juvenile tegu lizards in winter, when resting rates of oxygen consumption are nearly temperature-insensitive over the range of 17–25 °C. Such a degree of metabolic depression is comparable to that of small hibernating mammals that go into deep torpor when body temperatures drop to near 0 °C^23^. Thus, it is relevant to investigate the adjustments of the redox metabolism in the transitions from activity to dormancy, and back to full activity, in tegu lizards.

Among the tissues affected by metabolic depression events, the intestine has been studied mainly because of its poor perfusion during torpor^24^, its important contribution to the standard metabolic rate^25^, and its remarkable resistance to ischemia/reperfusion-derived oxidative stress in hibernators^26^. Nevertheless, the programed fasting during dormancy should elicit adaptations in this highly specialized absorptive tissue, whose function is expected to be strongly suppressed in the torpid phase and resume as soon as animals arise. From the redox point of view, there is evidence of increased lipid peroxidation and redox imbalance in the gut of squirrels during hibernation, which is related to low tissue perfusion and/or reperfusion during multiple torpor-arousal cycles^27,28^. Specifically in tegu lizards, the intestine shows a 50% reduction in the activities of citrate synthase and β-hydroxyacyl-CoA dehydrogenase, proxies of aerobic metabolism, as well as mucosal atrophy during dormancy^29^. The decrease in the heart frequency of approximately 80%^19^, together with the mucosal atrophy^29^ would lead to a substantial reduction of the blood flow in the organ. Furthermore, the reactivation of metabolism from torpor in juvenile tegus occurs gradually during arousal^20^, and it can take days for the tissue to resume its absorptive capacity^29^.

Considering the above mentioned metabolic adjustments that accompany the seasonal adaptations in the juvenile tegu intestine, we decided to investigate the redox metabolism under subtropical hibernation and the recovery from it in spring. The study was conducted during the first annual cycle of tegus to verify how early potential adjustments in antioxidant capacities manifest in the life cycle. The activity of antioxidant enzymes, the levels of glutathione and makers of oxidative stress (lipid peroxidation and protein oxidation) were determined during the lizard hibernation cycle. We also determined the alterations in the redox metabolism during fasting (for 20 days) in active lizards during spring, aiming to distinguish specific responses of metabolic depression during winter dormancy from the effects of food deprivation.

## Results

To assess the effect of subtropical hibernation and food deprivation on the intestinal redox metabolism of juvenile tegus, we measured the activities of antioxidant enzymes as well as accessory enzymes in four experimental groups, each one corresponding to a distinct stage of the first annual cycle. The activity of the H_2_O_2_-decomposing enzymes catalase and selenium-dependent glutathione peroxidase (SeGPX) did not change significantly in any group or after the 20-day fasting period, ranging from 29 to 44 U/mg protein, and from 42 to 71 mU/mg protein, respectively (Table 1). Similarly, the enzymes that compose the glutathione redox system, GR and G6PDH, were unaffected by either hibernation or food deprivation and showed activities ranging from 61 to 76, and from 3.4 to 4.2 mU/mg protein, respectively (Table 1).

**Table 1:** Activities of antioxidant and accessory enzymes in the intestine of tegu lizards under different metabolic states.

Metabolic state	Catalase (U/mg prot.)	SeGPX (mU/mg prot.)	GR (mU/mg prot.)	G6PDH (mU/mg prot.)
Late autumn	43.65 ± 3.85	70.91 ± 14.03	66.26 ± 3.47	3.49 ± 0.45
Winter dormancy	34.51 ± 4.89	67.01 ± 12.35	61.22 ± 5.06	3.44 ± 0.30
Arousal	32.27 ± 4.84	70.45 ± 6.97	64.14 ± 4.69	3.75 ± 0.57
Spring activity	42.05 ± 5.67	42.17 ± 4.43	76.03 ± 5.95	3.58 ± 0.64
Food deprivation	28.89 ± 3.32	46.97 ± 6.45	68.27 ± 1.48	4.17 ± 0.65

N = 7–9.

### Antioxidant status during the gradual metabolic depression in late autumn

**Figure 1:**
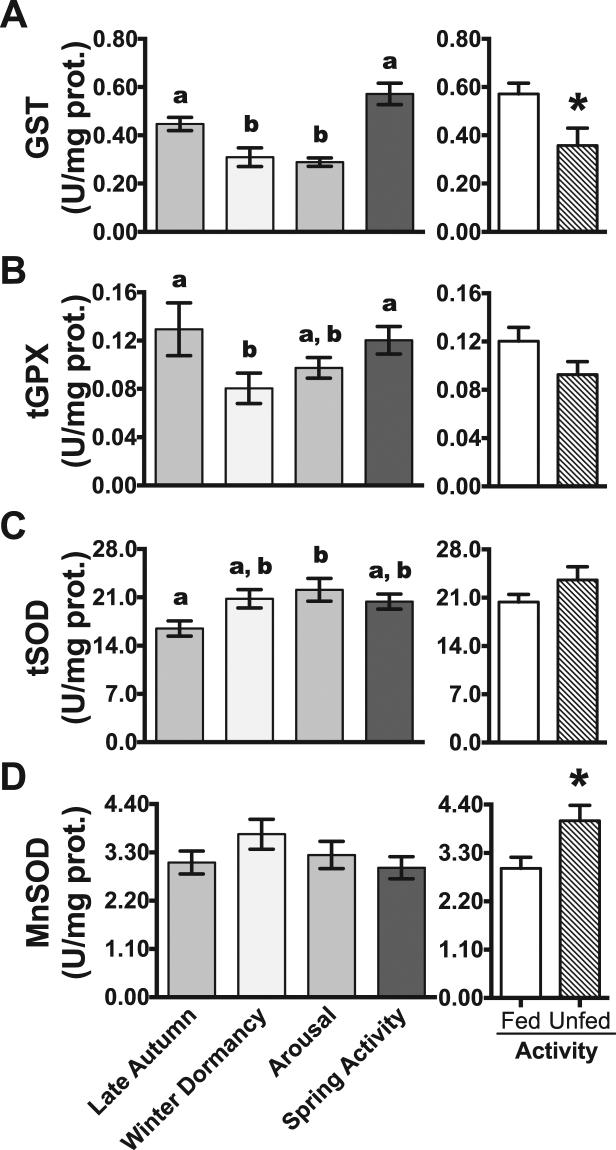
Antioxidant enzymes activities in the intestine of tegu lizards at different metabolic states and under food deprivation. (**A**) Glutathione transferase (GST), (**B**) total glutathione peroxidase (tGPX), (**C**) total superoxide dismutase tSOD (total superoxide dismutase) and (**D**) manganese superoxide dismutase (MnSOD). N = 7–8. Values are shown as mean ± s.e.m. ‘Spring activity’ and ‘Fed Activity’ data are from the same animal group. Different letters indicate significant differences between groups (P < 0.05). Asterisks indicate significant differences between fed and unfed active lizards (P < 0.05).

**Figure 2:**
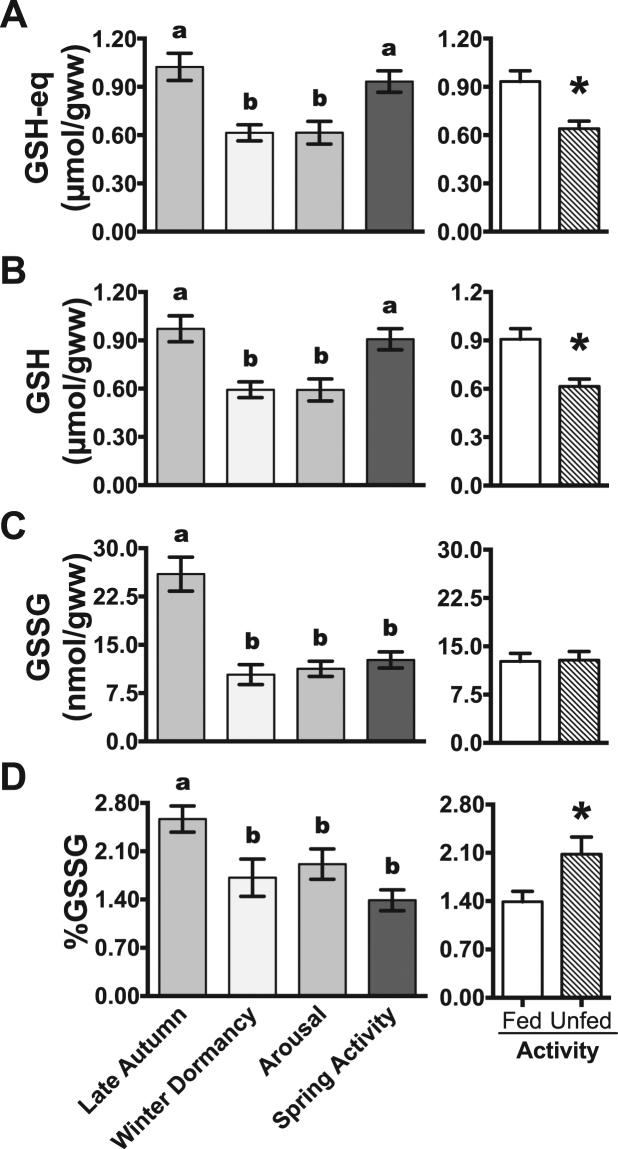
Redox status in the intestine of tegu lizards at different metabolic states and under food deprivation. Glutathione parameters: (**A**) Total glutathione equivalents (GSH-eq), (**B**) reduced glutathione (GSH), (**C**) disulfide glutathione (GSSG), and (**D**) GSSG/GSH-eq ratio (%GSSG). N = 6–8. Values are shown as mean ± s.e.m. ‘Spring activity’ and ‘Fed Activity’ data are from the same animal group. Different letters indicate significant differences between groups (P < 0.05). Asterisks indicate significant differences between fed and unfed active lizards (P < 0.05).

**Figure 3:**
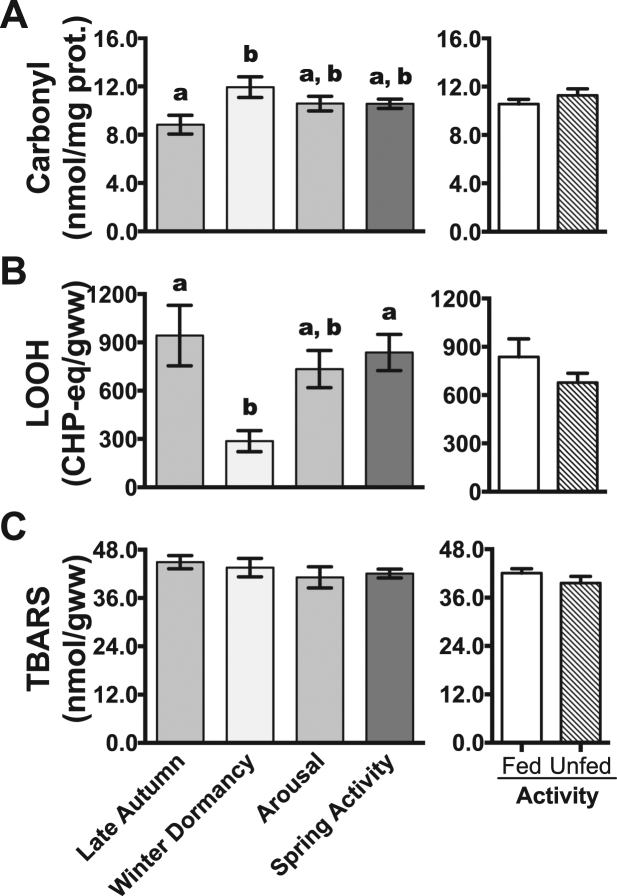
Oxidative stress markers in the intestine of tegu lizards under different metabolic states and food deprivation. (**A**) Carbonyl proteins (Carbonyl), (**B**) lipid hydroperoxides (LOOH), and (**C**) thiobarbituric acid reactive substances (TBARS). N = 5–8. Values are shown as mean ± s.e.m. ‘Spring activity’ and ‘Fed Activity’ data are from the same animal group. Different letters indicate significant differences between groups (P < 0.05).

Despite the partial reduction in the oxygen consumption rates^20^, the levels of all measured antioxidant parameters in the small intestine were similar between late autumn lizards (entering dormancy) and spring active lizards. The activities of GST (Fig. 1A), tGPX (Fig. 1B), tSOD (Fig. 1C) and MnSOD (Fig. 1D) were 0.45, 0.13, 16.48, and 3.07 U/ mg protein, respectively, in the intestine of late autumn animals. Such activities were not significantly different from those of spring active lizards. Similarly, total GSH-eq (Fig. 2A) and GSH (Fig. 2B) concentrations, as well as the levels of three oxidative stress markers (Fig. 3), were similar between late autumn and spring active lizards. On the other hand, GSSG concentration (Fig. 2C) and GSSG/GSH-eq ratio (Fig. 2D) were highest in animals entering dormancy during late autumn. Disulfide glutathione level and GSSG/GSH-eq ratio were 2.1 and 1.8-fold higher, respectively, in late autumn than in spring active lizards (Fig. 2C and D).

### Glutathione-dependent enzymes and oxidative stress markers during deep metabolic depression in winter

During winter, GST (Fig. 1A) and tGPX (Fig. 1B) activities decreased by 31% and 38%, respectively, in dormant lizards compared with late autumn lizards. Total and mitochondrial SOD activities remained unchanged in winter dormant lizards (Fig. 1C and D). Compared with late autumn lizards, winter dormant lizards had decreased levels of GSH-eq (by 40%), GSH (by 39%), GSSG (by 60%) and GSSG/GSH-eq (by 33%) (Fig. 2). Levels of TBARS did not change significantly throughout the hibernation cycle and they ranged from 44.9 nmol/g in late autumn to 41.1 nmol/g in arousal group. The content of carbonyl proteins was 35% higher after ~60 days of winter (approximately two thirds of the total hibernation period) compared to carbonyl levels in late autumn, (Fig. 3A). On the other hand, lipid hydroperoxides (LOOH) levels dropped from 942 nmol/g in late autumn to 286 nmol/g in winter dormancy, a fall of 70% (Fig. 3B).

### Partial recovery of the antioxidant capacity during arousal in spring

During the first 48–96 h of arousal, GST activity remained 35% lower than in late autumn lizards (Fig. 1A); tGPX activity returned to the levels observed in late autumn lizards (Fig. 1B); tSOD activity increased by 34% in comparison to late autumn lizards (Fig. 1C); and MnSOD activity did not change significantly (Fig. 1D). The levels of glutathione parameters GSH-eq, GSH, GSSG and GSSG/GSH-eq remained 40%, 39%, 57%, and 25% lower, respectively, during the first days of arousal in comparison to late autumn lizards (Fig. 2). Levels of carbonyl protein and LOOH returned to late autumn levels and TBARS concentration remained constant in the arousal group compared to the late autumn group (Fig. 3).

### Effect of food deprivation in spring active lizards

Food deprivation did not affect significantly the activities of tGPX (Fig. 1B) and tSOD (Fig. 1C), as well as the levels of three oxidative markers (carbonyl proteins, lipid hydroperoxides and TBARS; Fig. 3). Similar to winter dormancy, food deprivation elicited reductions in GST activity by 37% (Fig. 1A), in GSH-eq levels by 31% (Fig. 2A) and in GSH concentration by 32% (Fig. 2B). The lack of food did not affect GSSG levels in spring active lizards (Fig. 2C). On the other hand, unfed spring animals showed increased MnSOD activity (Fig. 1D) and GSSG/GSH-eq ratio (Fig. 2D), by 37% and 50% respectively.

## Discussion

In this study, we investigated the effects of subtropical hibernation and food deprivation on the redox metabolism in the intestine of juvenile *S*. *merianae*, an animal that does not exhibit multiple torpor-arousal cycles (typical of small mammals^19,23^) and hibernates at mild winter temperatures. Previous study had shown that in the transition from early to late autumn juvenile tegus reduce their resting metabolic rate at 25 °C by 51% (from 0.63 to 0.31 mL O_2_ h^−1^ g^−1^)^20^. During winter, metabolic rate, measured at 17 °C, drops further to a minimum of 0.12 mL O_2_ h^−1^ g^−1^ ^20^. Arousal from dormancy is gradual (lasting 3–5 days), and tegu metabolic rate at 25 °C increases progressively until it returns to the values of early autumn in spring activity (0.64 mL O_2_ h^−1^ g^−1^)^20^. The levels of GSSG/GSH-eq reached the highest values in the gut of late autumn lizards when compared to all other groups analyzed. Such high ratio indicates the occurrence of a redox imbalance in the intestinal tissue of late autumn lizards. In winter dormancy, GSH-eq and GSH levels decreased together with reductions in tGPX and GST activities, and in lipid hydroperoxides concentration. Overall, these effects could be associated with the prolonged fasting and metabolic depression in winter. Moreover, arousal from dormancy did not induce redox imbalance or oxidative stress in the intestinal tissue even though antioxidant defenses were maintained or reduced in winter.

Food deprivation during spring activity disturbed the redox balance in the intestine as a result of the diminished GSH levels, indicating that the cause of the redox imbalance would be different from that during the late autumn. On the other hand, the interruption of food intake in spring-activity tegus led to effects that are similar to those in winter dormancy in relation to changes in GSH levels and GST activity, although the redox balance was maintained during winter dormancy. These contrasting results highlight the importance of anticipatory adjustments, which prepare the intestinal tissue for the prolonged winter fasting. Fittingly, food deprivation in spring-active animals resulted in intestinal morphological changes that differ from those observed in fasting during winter-hibernation^29^.

Glutathione is an abundant low molecular weight thiol that acts as a redox buffer, and its disulfide form is reduced by GR at the expense of NADPH. The ratio GSSG/GSH-eq is a reliable predictor of the cellular redox balance^30^. Tegu metabolic state prior to dormancy was associated with a redox imbalance, as indicated by the two-fold higher GSSG levels and GSSG/GSH-eq ratio in late autumn compared to all other groups, including spring activity animals, although the activities of the glutathione redox-system enzymes (GR and G6PDH) remained unaffected. Similar results were observed in the intestine of 13-lined ground squirrels during entrance into torpor, when the redox-sensitive transcription factor NF-κB is activated, and the GSSG/GSH-eq ratio, as well as GSSG concentration rise compared with active squirrels^27,28^. In another model of metabolic depression, GSSG levels increase, resulting in a higher GSSG/GSH-eq ratio, in the intestine of estivating spadefoot toads compared with active animals^31^. Similarly, GSSG concentration and GSSG/GSH-eq ratio both increase in muscle of lungfish during the first days of estivation^32^. Furthermore, an increase in GSSG concentration also occurs in tissues of estivating land snails^33^ and in tissues of hibernating frogs from Tibet mountains^11^.Therefore, a redox imbalance may occur prior to and/or during depressed metabolic states in animals. Even though the tegu intestine experienced a more oxidized state during late autumn, this was not enough to inflict significant oxidative damage (as protein oxidation or lipid peroxidation).

**Figure 4:**
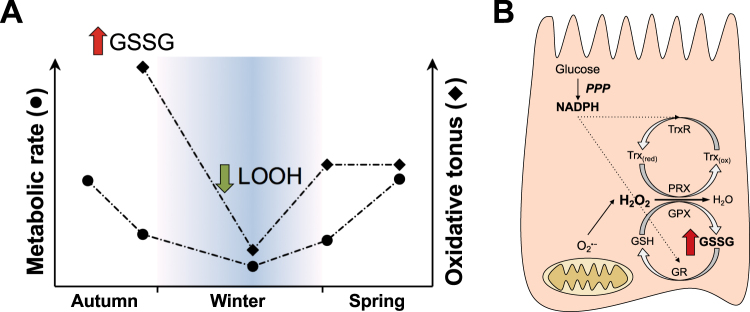
Schematic model illustrating hypothetical changes of the ‘oxidative tonus’ in the small intestine of juvenile tegu lizards, associated with seasonal changes in metabolic rate during their first annual cycle. (**A**) Circles depict the decrease of resting metabolic rates during the autumn and winter in neonate tegus, and the reactivation during arousal in spring (data from de Souza *et al*., 2004). Diamonds depict the ‘oxidative tonus’ of the intestinal tissue in the corresponding stages of the annual cycle, according to the current results. The tegus hatched during the summer, and early autumn samples were not available for this study. Late in the autumn, the highest GSSG levels indicate a redox imbalance and a more oxidized environment in intestinal tissue. During winter dormancy the concentration of LOOH decreased, indicating that proportional adjustments in the processes that produce and consume energy promote a lower steady state concentration of ROS. The reactivation of metabolism is gradual during arousal from dormancy, restoring the pre-hibernation redox balance in the intestinal tissue. (**B**) Hypothetical mechanism to explain the increased GSSG levels observed during late autumn in the tegu intestine. NADPH should be generated in large amounts through the pentose phosphate pathway (PPP) in the intestinal mucosa of actively growing tegus (in summer and early autumn). However, in late autumn metabolic rates slow down and NADPH supply should – hypothetically – decrease in the tissue. This may impair the enzymatic management of H_2_O_2_ (mainly by GPX and peroxiredoxins), leading to redox imbalance. LOOH, lipid hydroperoxides; GPX, glutathione peroxidase; GR, glutathione reductase; GSH, reduced glutathione; GSSG, disulfide glutathione; PRX, peroxiredoxin; Trx_(ox)_, oxidized thioredoxin; Trx_(red)_, reduced thioredoxin; TrxR, thioredoxin reductase.

Possible causes of elevated GSSG levels include (*i*) an inability to recycle GSSG back to GSH, and (*ii*) an increase in ROS production. In late autumn tegus, the activities of GR and G6PDH in the intestinal tissue were unaffected (indicating a sustained potential ability to recycle GSH), when whole body resting metabolic rate is 51% lower than in early autumn tegus (but it is still higher than that of winter dormant lizards^20^). A hypothesis that would fit this setting is a lowering of reducing potential (i.e. NADPH) availability to keep GSH recycling (Fig. 4). In the case of juvenile lizards, they must balance the use of NADPH to grow/develop, build energy reserves and control redox balance. During this period, despite the drop in the resting metabolic rate in relation to early autumn^20^, lizards still ingest some food and the absorptive capacity of the small intestine and the activities of key metabolic enzymes are well preserved^29^. NADPH is generated in considerable amounts through the pentose phosphate pathway in proliferative tissues as the intestinal mucosa. However, given the condition of intermittent feeding and the concurrent demand for reducing potential from anabolic pathways, as animals grow and store reserves, this fundamental source of reducing equivalents (which is NADPH) might become limited in tegu intestines during entry into dormancy in late autumn. With a hypothetically diminished NADPH availability, the management of intracellular ROS, especially H_2_O_2_, by the NADPH-dependent GSH/GPX/thioredoxin/peroxiredoxin antioxidant systems should be hindered, leading to GSSG accumulation and redox imbalance (Fig. 4B). Thus, based on the above assumptions, the increase in GSSG levels in the tegu intestine is not likely a consequence of an increased rate of ROS formation, but a result of decreased *in situ* antioxidant capacity, in agreement with the “redox-optimized ROS balance” hypothesis^34,35^. This hypothetical decrease in antioxidant capacity *in situ* is independent on the maximal activities of antioxidant enzymes measured in gut, which were preserved in late autumn. The dependence of intestinal antioxidant activity on NADPH supply was observed in rats, in which enterocytes decompose t-butylhydroperoxide at half speed comparing fasted with fed animals^36^. Such limited ability to eliminate reactive species is fully reverted by the addition of glucose^36^. In the case of tegus, it is still necessary to determine gut NADPH levels – and other determinants of redox control, such as thioredoxin – to further understand why the tissue develops an increased oxidative state in late autumn.

In winter-dormant tegus, as well as in food deprived active animals, the decreased nutrient supply is expected to affect glutathione synthesis, an ATP expensive process that, in the intestine, depends on the luminal (dietary) or blood (e.g. hepatic) supply of amino acids^37^. Both the total glutathione pool and GSH levels fell significantly in winter dormant lizards compared to either late autumn or spring active animals. Depletion of hepatic GSH levels in food deprived animals have been reported for fish^38^, rats^39–42^, mice^43^ and guinea pigs^44^. One consequence of such glutathione depletion is a higher susceptibility to ischemia-reperfusion derived oxidative damage in fasted rats compared to those fed normally^45^. Similar to liver, GSH content also decreases in small intestine of food deprived animals^46,47^, making it more susceptible to oxidative challenge^48^. In tegus, although levels of GSH (essential for the enzymatic action of GPX and GST) decreased, oxidative damage was either unchanged in food deprived animals or without major effect in dormant ones. In winter dormant tegus, markers of lipid peroxidation were either highly depressed (LOOH) or unchanged (TBARS), while carbonyl protein levels slightly increased (see Fig. 3). Moreover, the decrease in %GSSG – as well as GSSG levels – is evidence of the overall reduction in ‘oxidative tonus’ in dormancy (Fig. 4A). Under deep winter-dormancy, it is expected that mitochondrial ROS formation – and therefore the induction of lipid peroxidation – is much decreased due to the overall 80% reduction in metabolic rate. Accordingly, key metabolic pathways in intestine are suppressed and intestinal tissue atrophy occurs in this period^29^. From early autumn to the onset of arousal, in early spring, the intestine shrinks from 1% of the tegu body mass to 0.77%^29^, analogous to the observed in fasting or estivating anurans^49,50^. In addition, the gastrointestinal tract is in direct contact with the environment and must deal with potentially harmful oxidants and electrophiles^51,52^ and different GST isoforms are responsible for the detoxification of xenobiotics from food^53^. Therefore, as another consequence of the interruption of food intake, the exposure of the gut tissue to xenobiotics is much reduced or absent. Thus, the decrease in GSH levels and GST activity may be related to the lack of food for both winter dormant and unfed lizards. In rats, for instance, fasting results in decreased intestinal GST activity^54^. Moreover, during winter dormancy, the decreased metabolic rate may slow down the energy expensive process of GSH biosynthesis, leading to a decrease in total glutathione levels.

Carbonyl proteins are stable products of protein oxidation by reactive species that are widely used as markers of oxidative damage^55^. Carbonyl proteins levels were higher after ~60 days of winter dormancy in relation to levels in late autumn, when lizards were at the onset of hibernation, The 20S proteasome recognizes and degrades oxidatively modified proteins (e.g. protein containing carbonyl groups) in an ATP- and ubiquitin-independent manner^56^. The increased steady state of carbonyl protein level in tegus during winter can be viewed as the result of two opposing processes: increased oxidative damage to proteins (even though ROS levels are possibly smaller in dormancy), reduction in the degradation of oxidized proteins (due to suppression of proteasome 20S activity), or a combination of both. In torpid golden-mantled squirrels, hepatic 20S proteasome activity does not differ from summer active squirrels when assayed at the same temperature^57^. Such activity was found to be much lower when assays were conducted at temperatures commonly recorded in the body of torpid squirrels^57^. Furthermore, the rate of protein renewal by *de novo* synthesis is expected to be reduced by the state of limited energy and lower temperature during winter dormancy^58^. Thus, the decrease in temperature during winter could account for the higher carbonyl content in gut during tegu dormancy. On the other hand, during spring arousal, there is a reversal of the effects that promoted carbonyl protein accumulation in winter, which is consistent with the gradual morphological rearrangement and metabolic reactivation in the tegu gut tissue^29^. The levels of redox parameters that decreased during winter – lipid hydroperoxides, GSH and GST – returned to previous levels during arousal before food intake. Interestingly, key metabolic enzymes are still low during early-arousal (their activities are similar to those in the winter^29^) and whole body metabolic rate is still at half of the full spring activity^20^.

Unfed active lizards during spring showed increased MnSOD activity and increased GSSG/GSH-eq ratio in the intestine. Different from the gradual decrease of food intake in late autumn, for which animals prepare themselves, food deprivation in spring represents an unanticipated event. While in late autumn the augmented GSSG/GSH-eq ratio was related to increased GSSG levels, in food-deprived lizards the increased ratio was related to decreased GSH concentration. Thus, the two events of redox imbalance are caused by different phenomena. Additionally, the increase in MnSOD activity indicates the occurrence of a mild redox imbalance in food deprived tegus, which was not associated with significant changes in other oxidative stress markers. Similar responses were observed in nutrient-starved and oxidatively challenged cells. For instance, mice exposed to caloric restriction show increased MnSOD activity in white adipose tissue associated to the deacetylation of specific lysine residues^59^. The activation of endogenous antioxidants in response to fasting has been reported for several species. For instance, fasting leads to increased SOD activity in the plasma of emperor penguins^60^, increased hepatic CuZnSOD expression (at both mRNA and activity levels) in yellow croakers^61^, and increased total SOD activity in liver and gills of brown trout^62^.

Events of redox imbalance and oxidative stress are expected to prompt increases in activity/expression of antioxidant enzymes, mediated by redox sensitive transcription factors, such as Nrf2^63^ and NF-κB^64^, as observed in tissues of many hibernating^8,65,66^ and estivating animals^31,33,67^. For instance, in torpid 13-lined ground squirrels, the GSSG:GSH ratio in intestinal mucosa is 5-fold higher than in summer animals, an effect due primarily to elevated GSSG concentrations during hibernation^28^. Additionally, nuclear translocation of NF-κB is greater in intestinal mucosa from hibernating compared with summer animals, but differences exist during torpor-arousal transitions since activation of NF-κB is highest as animals enter torpor, remains high throughout a torpor bout, and is lowest in hibernators arousing from torpor^27^. In the case of the intestine of juvenile tegu lizards, the high GSSG concentration observed in late autumn was not accompanied by increases in antioxidant defenses as happens in many animal species employing the physiological mechanism of “preparation for oxidative stress” (POS)^2,68^. On the contrary, the antioxidant capacity was down in winter dormancy, as well as in the previous pre-hibernating stage (late autumn – Fig. 4A). Our results resemble recent observations that winter-dormant frogs *Nanorana parkeri* from the Tibet high mountains present decreased antioxidant enzyme activities in various organs when compared to summer-animals^11^. Even though the POS mechanism is employed by a large proportion of animal species whose redox metabolism was studied, 30% of the analyzed vertebrates are POS-negative species^68,69^. Thus, based on the several parameters measured herein, the intestine of juvenile tegu lizards can be classified as POS-negative during dormancy. The measurement of other components of the antioxidant system (e.g. peroxiredoxins) and/or other tissues may change this classification in the future.

Our results indicated the lack of increased signs of oxidative stress (except for carbonyl protein levels) nor activation of endogenous antioxidants during subtropical winter dormancy in the intestine of juvenile tegus. This happened in the transition from autumn (21–26 °C in early season, inside tegu cages) to winter (15–20 °C). The 80% decrease in metabolic rate in dormancy is accompanied by a drop in ‘oxidative tonus’, a condition where activity/levels of endogenous antioxidants are diminished as well as – putatively – ROS formation. Conversely, markers of oxidative stress (GSSG, carbonyl protein and lipid peroxidation) increased in various organs of *N*. *parkeri* frogs during dormancy^11^. Moreover, there was no statistically significant evidence of oxidative stress during arousal from hibernation in juvenile tegus. This contrasts with Arctic ground squirrels, where the quick return to 37 °C is accompanied by increased markers of oxidative stress in liver and brown adipose tissue^70^. This could be related to the slow return of metabolic rates to the levels of full activity, while in ground squirrels it takes only few hours. Whether or not mature tegus undergo oxidative stress during post-hibernating arousal, which involves thermogenesis^71,72^, is an issue for future investigation.

Furthermore, the regulatory mechanisms that affect the activities of antioxidant enzymes in a seasonal basis (winter hibernation *versus* spring activity, for example) are largely unknown. Our results indicated differences in the V_max_ at 25 °C for selected enzymes in homogenates, suggesting non-thermal seasonal effects on redox metabolism in the juvenile tegu small intestine. The observable effects may be due to variation in enzyme concentration (by changes in either transcription and/or translation rates), occurrence of post-translation modifications, or alterations in the relative abundance of allozymes and isozymes with varying affinities for substrates^73^. The understanding of such mechanisms, as well as the effect of temperature on the kinetics of these antioxidant enzymes constitute an interesting scenario for further steps of investigation.

## Methods

### Animal rearing

Juvenile tegu lizards (*Salvator merianae*, formerly *Tupinambis merianae*) were obtained from a population reared outdoors in large enclosures at the State University of São Paulo in Rio Claro, Southeastern Brazil, under the permit for rearing animals from IBAMA 02001-000412/94-28. Two months after hatching in the summer, juvenile lizards were transferred to other laboratory facilities at the University of São Paulo in São Paulo, where they were used in experiments during the first annual cycle^20,74^. The animals used in this study originated from two clutches of eggs collected in 2007, and all tissue samples were taken during the first annual cycle. Briefly, groups of 5–6 lizards were kept in several 150-liter plastic cages, and an incandescent lamp provided light and heat 8 hours a day (8:16, light:dark). A wooden shelter in one extremity, and stones positioned under the lamp in the other, allowed the animals to freely move to cooler or warmer places inside the cage. Additionally, the cage lid allowed the diffusion of natural sunlight and air coming through the window from the outside environment. During autumn, the tegus emerge later and retreat earlier so that the time devoted to thermoregulation becomes progressively shorter, until the lizards become continuously inactive inside the shelter. The tegus were kept in the shade through the winter and were returned to the previous photo- and thermal periods after arousal in early spring. The temperature mean ranges inside the cages were: early autumn, 21–26 °C; late autumn, 18–23 °C; winter, 15–20 °C; early spring, 20–26 °C; late spring, 23–30 °C. The minimum values are nighttime air temperatures recorded in the shelter area, and maximum values are daytime air temperatures recorded in the stone area. Animals were fed every two days on raw meat, eggs and fruits and had continuous access to drinking water. As the animals naturally stop eating, feeding was interrupted during the inactivity phase and restored 48 h after arousal. During the first days of arousal the tegus were still anorexic and drink water abundantly, and a gradual increase in food intake took place over the following days.

As the annual cycle progressed towards winter, the tegus changed their behaviors as detailed previously^20^, and a defined set of behaviors was used to classify animals in each of the following four groups. In the ‘late autumn’ group (late May), animals were leaving their cages later to thermoregulate and sporadically feed during the day, however, they had lowered their locomotor activity and exhibited behaviors peculiar to the onset of hibernation. In the ‘winter dormancy’ group (late August; around 2/3 of the total hibernation period) they had been totally inactive for 50–60 days during the winter. Tegus of the ‘arousal’ group (late September) had aroused from dormancy for 48–96 h, were rehydrated, but did not feed yet. In the ‘spring activity’ group (late October), animals had resumed completely their activities (locomotion, feeding and growth) for 30–40 days during spring. One additional group, named ‘spring activity – unfed’, was composed of animals that were food deprived for 20 days, with access to water. The 20-day length of food deprivation was chosen because it would demand a comparable amount of endogenous energetic reserves to support the metabolism during winter dormancy in hibernating tegus.

The small juvenile lizards were decapitated, and the head and trunk were immediately immersed in ice for quick dissection of the intact brain, the gut, and other organs to use in different studies. Anesthetics were not used prior to decapitation aiming for tissue samples free of chemicals. In an attempt to reduce distress, the procedure was always performed in the morning before the lizards warm up and raise their metabolic rate and activity. The middle portion of the intestine was dissected, rinsed in physiological saline solution, blotted on paper, weighted, frozen in liquid nitrogen and stored at −80 °C until assayed. All laboratory work was done before the approval of the Brazilian Federal Law 11,794, regulating the creation of institutional Ethics Committees on animal use for teaching or scientific purposes. The institutional committees became mandatory in 2009, after the work was concluded (in 2008). Nevertheless, the animal care and euthanasia procedures followed ethical principles in animal experimentation described by the U.S. ‘Guide for the Care and Use of Laboratory Animals’ and ‘AVMA Guidelines on Euthanasia’ available at the time the work was performed. Animal care and euthanasia procedures were approved (in February 2018) by an independent veterinary from the University of Brasilia.

### Activities of antioxidant and related enzymes

Intestine samples were homogenized in ice-cold 50 mmol l^−1^ potassium phosphate buffer pH 7.2 containing 0.5 mmol l^−1^ EDTA and 0.1 μg/g of tissue wet weight phenylmethylsulfonyl fluoride (added just prior to homogenization). Homogenates were collected and centrifuged at 10,000 × g for 15 min at 4 °C. Supernatants were transferred to new tubes and stored on ice for further use in the assays. The activities of the main antioxidant enzymes, catalase, total glutathione peroxidase (tGPX), selenium-dependent glutathione peroxidase (SeGPX), glutathione transferase (GST), glutathione reductase (GR), glucose 6-phosphate dehydrogenase (G6PDH), and total superoxide dismutase (tSOD) were measured spectrophotometrically as recently described^75^. To assay manganese superoxide dismutase (MnSOD) activity, samples were prepared using the same protocol for tSOD, but with the addition of 2 mmol l^−1^ potassium cyanide (to inhibit CuZnSOD). Enzymatic activity are expressed in relation to the soluble protein content, which was measured by reacting supernatants with Coomassie brilliant blue G-250 and comparing them to a standard curve built with bovine serum albumin^76^.

### Glutathione parameters and carbonyl protein

Frozen tissues were homogenized in ice-cold 10% (w/v) trichloroacetic acid with a hand-held glass homogenizer. The crude homogenate was centrifuged 10,000 × g for 6 min at 4 °C. The supernatant was used for the determination of glutathione parameters and the pellet used for the protein carbonyl assay. Total glutathione equivalents (GSH-eq), reduced glutathione (GSH), disulfide glutathione (GSSG), and the ratio between disulfide and total glutathione (GSSG/GSH-eq) were measured by the enzymatic recycling method as described previously without any modification^75^. Glutathione parameters are presented as μmol or nmol per gram of wet weight. The levels of protein carbonyls were quantified as an index of protein oxidation by ROS following a spectrophotometric protocol previously described^75^. Carbonyl concentration is expressed as nmol per milligram of protein.

### Lipid peroxidation markers

Two biochemical indicators of lipid peroxidation were measured. The thiobarbituric acid reactive substances (TBARS) assay was used to detect lipid peroxidation end products, whereas the xylenol orange assay was used to estimate the levels of lipid hydroperoxides (LOOH), which are intermediate products of the lipid peroxidation cascade. For the xylenol orange assay, tissues were homogenized in ice-cold methanol using a hand-held glass homogenizer. The resulting homogenate was centrifuged for 5 minutes at 10,000 × g and 4 °C, then the supernatant was used in the assay following procedures as previously described^77^. LOOH content is expressed as cumene hydroperoxides equivalents per gram of wet weight (CHP-eq/gww). Phosphoric acid at 1.1% (w/v) containing 0.1 mmol l^−1^ butylated hydroxytoluene was used to homogenize intestine samples in a hand-held glass homogenizer in ice. The crude homogenate was directly used in the TBARS assay as previously described^33^. Levels of TBARS are expressed as nmol per gram of wet weight.

### Statistical analysis

Prior to the application of parametric or nonparametric statistical tests, Shapiro–Wilk and Levene’s tests were performed to evaluate the assumptions of normality and homogeneity of variance respectively. Then, one-way ANOVA (or nonparametric Kruskal Wallis test) was used to compare ‘late autumn’, ‘winter dormancy’, ‘arousal’ and ‘spring activity’ groups, followed by the post hoc Tukey test (or nonparametric Mann-Whitney test). To evaluate the effect of food deprivation in active lizards during the spring, ‘fed’ and ‘unfed’ groups were compared by means of parametric two-tailed Student’s t-test or nonparametric Mann-Whitney test. All statistical analyses were conducted using SPSS (version 13.0, Chicago, SPSS Inc.), and the tests assumed *P* values below 0.05 to be statistically significant. Data are presented as mean ± s.e.m. The datasets generated during and analyzed during the current study are available from the corresponding author on reasonable request.
